# Development and evaluation of a training workshop for lay health promoters to implement a community-based intervention program in a public low rent housing estate: The Learning Families Project in Hong Kong

**DOI:** 10.1371/journal.pone.0183636

**Published:** 2017-08-25

**Authors:** Agnes Y. Lai, Sunita M. Stewart, Alice Wan, Helen Fok, Hebe Y. W. Lai, Tai-hing Lam, Sophia S. Chan

**Affiliations:** 1 School of Public Health, The University of Hong Kong, Hong Kong, SAR, China; 2 Department of Psychiatry, University of Texas Southwestern Medical Center at Dallas, Dallas, Texas, United States of America; 3 Christian Family Service Centre, Hong Kong SAR, China; 4 School of Nursing, The University of Hong Kong, Hong Kong, SAR, China; TNO, NETHERLANDS

## Abstract

This paper presents the development and evaluation of the train-the-trainer (TTT) workshop for lay resident leaders to be lay health promoters. The TTT workshop aimed to prepare the trainees to implement and/or assist in conducting a series of community-based family well-being activities for the residents in a public low rent housing estate, entitled “Learning Families Project”, under the FAMILY project. The four-hour TTT workshop was conducted for 32 trainees (72% women, 43% aged ≥ 60, 41% ≤ elementary school education). The workshop aimed to promote trainees’ knowledge, self-efficacy, attitude and practice of incorporating the positive psychology themes into their community activities and engaging the residents to join these activities and learn with their family members. Post-training support was provided. The effectiveness of the TTT was examined by self-administered questionnaires about trainees’ reactions to training content, changes in learning and practice at three time points (baseline, and immediately and one year after training), and the difference in residents’ survey results before and after participating in the community activities delivered by the trainees. The trainees’ learning about the general concepts of family well-being, learning family, leadership skills and planning skills increased significantly with medium to large effect sizes (Cohen’s d: 0.5–1.4) immediately after the training. The effects of perceived knowledge and attitude towards practice were sustained to one year (Cohen’s d: 0.4–0.6). The application of planning skills to implement community activities was higher at one year (Cohen’s d: 0.4), compared with baseline. At one year, the residents’ survey results showed significant increases in the practice of positive communication behaviours and better neighbour cohesions after joining the family well-being activities of LFP. Qualitative feedback supported the quantitative results. Our TTT workshop could serve as a practical example of development and evaluation of training programs for lay personnel to be lay health promoters.

**Trial registration:** ClinicalTrials.gov NCT02844244

## Introduction

We describe the framework, development and results of a systematic evaluation of a training workshop for lay resident leaders to be lay health promoters of a public low rent housing estate in Hong Kong. The training was delivered in two two-hour sessions, which was shorter than many programs described in the literature and therefore was less burdensome and cheaper. Our project is also unusual in that the trainees were older and less educated (42% aged ≥60, and 41% had only elementary education or less). The trainees were taught both to implement with guidance and to assist social workers to conduct a series of community-based family well-being activities, under ‘The Learning Families Project’ in Hong Kong [1]. The Learning Families Project was part of the project titled ‘FAMILY: a Jockey Club Initiative for a Harmonious Society’ [2]. The Learning Families Project aimed to involve trained lay community volunteers to enhance the residents’ family well-being, and neighbourhood cohesion through a series of community activities.

This collaborative project involved academic researchers from School of Public Health of The University of Hong Kong (HKU-SPH), social workers of Christian Family Service Centre (CFSC) and resident leaders of Estate Management Advisory Committee (EMAC) and Mutual Aid Committees (MAC) of Tsui Ping (South) Estate in Kwun Tong. CFSC is a non-government charitable organization focusing on the care and support to needy families. Kwun Tong District is one of the most impoverished districts in Hong Kong [3]. This community is marked by high poverty and domestic violence rates, and a large proportion of older people among its residents [3, 4]. Tsui Ping (South) Estate is a public housing estate with a population of 13400 residents in 5000 households, and was selected as the setting for this project because of its proximity to CFSC.

The train-the-trainer (TTT) model is gaining increasing attention as an effective strategy to build a community workforce for health promotion and disease prevention [5]. In contrast to the traditional model where experts deliver services, in the TTT model, experts train key stakeholders to deliver services. Training for participants from the same community they serve as lay health promoters (volunteers), can help build knowledge at the local level. Lay health promoters with training and supervision were shown to have significant impacts on community-based interventions [6, 7]. The TTT model has been applied to train lay health promoters to enhance physical activity [8–10], nutrition [10–12], eye health and safety [13], and heart health [14]. The training of lay health promoters and reliance on community resources simultaneously reduce the demand on time, resources and manpower from financially strapped and understaffed professional social health services in the community [15]. Our TTT workshop targeted lay resident leaders who lived in the same community they now served and would deliver the proposed activities after training. Because of their knowledge of the community, these trainees, if well trained, could communicate in a way that was more applicable, practical, and culturally appropriate [14, 16]. Consequently, community participants might be more likely to join the health promotion programs [8, 17], accept the health information and the values promoted [18] and change their behaviour [19].

**Fig 1:**
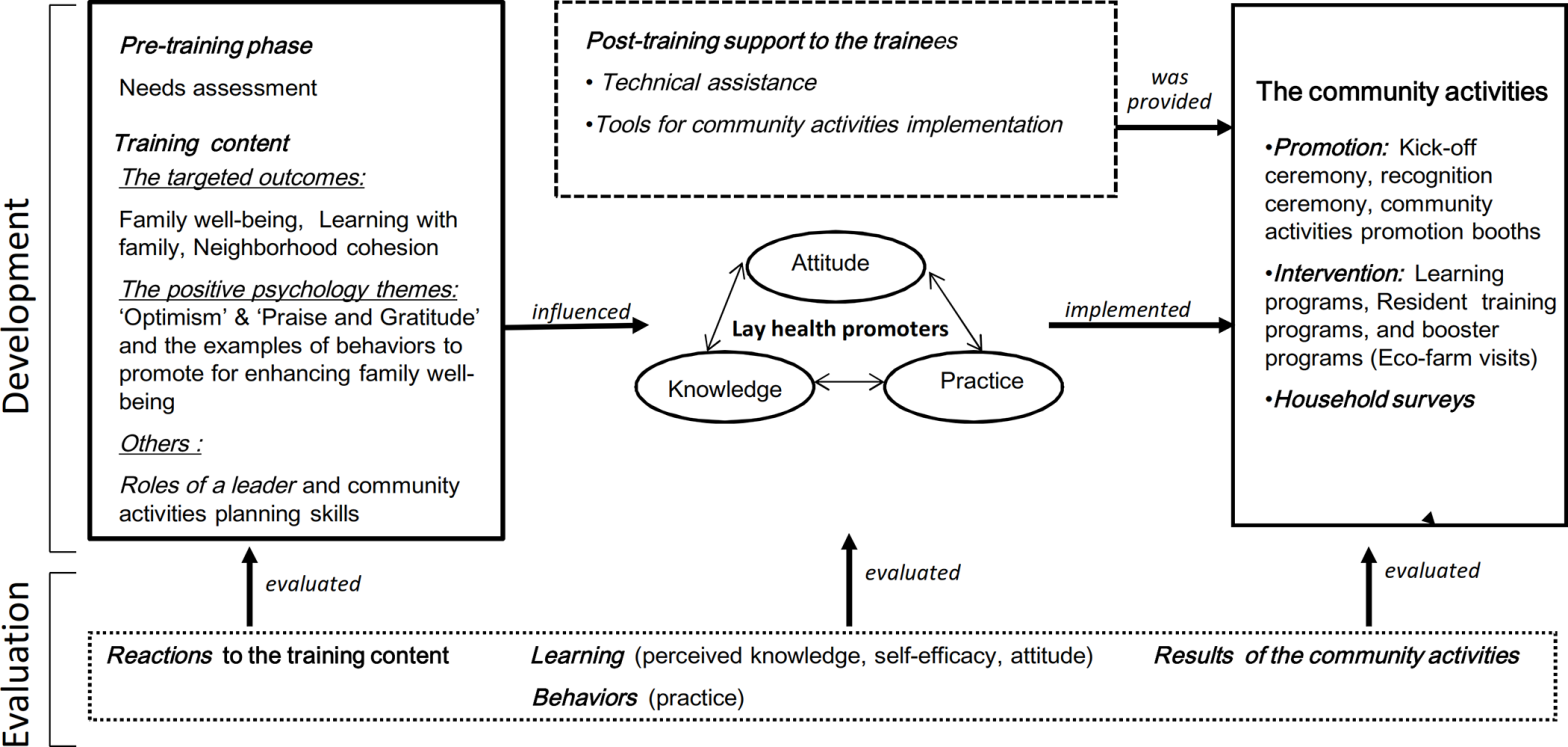
The framework for the development and evaluation of the train-the-trainer workshop to implement community intervention program.

The current TTT workshop focused on the enhancement of knowledge, attitude, and practice of trainees on implementing and assisting to conduct community activities for the residents. The development and evaluation of the TTT workshop was guided by a theory-based framework (Fig 1). We assessed the TTT workshop systematically, including the entire process of engagement, capacity building and subsequent intervention programs (community activities). Questionnaires were used to evaluate the effectiveness of the TTT workshop on four dimensions, adapted from the Kirkpatrick's Four-Level Training Evaluation [20]: (i) reactions to the training content; (ii) changes in perceived knowledge, self-efficacy, and attitude; (iii) changes in practice of applying what had been learned; and (iv) results of the community activities of this project implemented and/or assisted by the trainees. Semi-structured focus group interviews were utilized to explore the issues/difficulties the trainees faced when conducting the community activities. The primary outcome was change in trainees’ learning in developing and implementing the community activities for the residents. The secondary outcomes were the trainees’ reactions to training content, and the practice of implementing community activities, and the effectiveness of the trainees’ implemented community activities.

The current study could make some novel contributions to the literature on TTT workshop in that we illustrate the collaborative work of different stakeholders, and the formal evaluation on the development and effectiveness of the training with a long follow-up of one year.

## Materials and methods

### Study designs

This was a single-group evaluation study. All resident leaders of EMACs and MACs in Tsui Ping (South) Estate were invited to join the training. Inclusion criteria were the following: (i) Ethnic Chinese older 18 years of age, (ii) able to read Chinese and speak Cantonese, and (iii) willing to carry out the duties of lay health promoters in the project. The evaluation included quantitative questionnaire assessments at three time points: before training, immediately afterwards, and one year after training. In addition, qualitative focus group interviews were completed at 18 months after training. A needs assessment was conducted in November 2010; the TTT workshop started in Dec 2010; and the follow-up assessments and focus group interviews were completed in Dec 2011 and June 2012, respectively. Ethical approval was granted by Institutional Review Board (IRB) of the University of Hong Kong/Hospital Authority Hong Kong West Cluster (HKW IRB reference number: UW10-041). Written informed consents were obtained from the participants.

### The intervention

#### Pre-training phase

It has been proposed that community-based training programs should target the needs of the community, and have clear objectives to enable the community advance towards its goals [21]. We invited 16 resident leaders of EMAC and MACs to join focus group interviews, which aimed to: (i) understand their roles and responsibilities in Tsui Ping (South) Estate; (ii) collect the views of the resident leaders towards the general concept of learning with family members to enhance family well-being; and (iii) collect their suggestions for the design and implementation of the project. This information was important to frame the training objectives and guide the design and content of the training workshop. All who joined the interviews were invited to take on the role of lay health promoters for the project.

#### Training phase

The training workshop with two two-hour sessions was conducted by social workers (frontline service delivery professionals) and academic public health professionals (a doctor and a nurse) who were experienced in conducting community-based interventions. The aim was to enhance the trainees’ cognitive factors (knowledge, self-efficacy, and attitude) which influenced their competencies and behavior in implementing and/or assisting to conduct the community activity interventions. We designed a package of learning activities for the trainees to acquire the specific knowledge and skills they needed for the activities. Fig 1 lists targeted outcomes and training contents of the workshop, and post-training support to the trainees for implementing and assisting to conduct community activities. The training content, structure and methods, were simple, easy to understand and convenient to practice (Table 1).

**Table 1:** The training content of the train-the-trainer workshop.

Session One (2 hours)	Session Two (2 hours)
To introduce the key stakeholders of the project to each other (15 minutes)	To introduce the general concept of ‘Family well-being’ and ‘Learning Family’ (30 minutes)
To introduce the aims and conceptual framework of the project (30 minutes)	To discuss the importance of neighbouring relationship and neighbourhood cohesion (20 minustes)
To discuss the roles of leader and leadership skills (30 minutes)	To introduce the general concept and application skills of ‘Optimism’, and ‘Praise and Gratitude’ (30 minutes)
To illustrate the expected role of lay health promoters and the work plan of the project (20 minutes)	To introduce ‘5W2H’ planning skills for program planning (40 mintes)

The first session began with an ice-breaking game to introduce the key stakeholders of the project to each other. The stakeholders included the research team from HKU-SPH, social workers from CFSC, and resident leaders from different housing blocks in Tsui Ping (South) Estate. Then, we introduced and discussed the overall project aims and the conceptual framework. We explained the distinctiveness of the Learning Families Project in that the community was utilized as the setting, the target, the agent and the resources for the intervention [22]. We explained the roles of a leader and the duties of a lay heath promoter. The duties of a lay health promoter included: (i) the promotion of ‘Learning Families Project (for example, promotion activities, telephone calls, mobile counters, and door-to-door canvassing), (ii) the development and implementation of the community activities (for example: kick-off and recognition ceremonies, Learning programs, and Resident training programs), and (iii) the evaluation of the community activities (for example: household surveys and questionnaire assessments on the residents).

In the second session, we introduced the key components of the project, including the general concepts of ‘Family well-being’(2), ‘Learning family’ [1, 23], and ‘Neighborhood cohesion’ [24] (Table 2). The project was grounded on the concept of Learning family [1, 23], implied that family communication and family well-being can be promoted when family members participate in learning activities and learn something together. The residents were encouraged to join the community activities, including talks and day camps with their family members. These activities were designed to be enjoyable, while promoting family relationships and well-being, as well as neighborhood cohesion. The activities provided platforms for the residents to (i) communicate with their family members and neighbours, (ii) enjoy each other’s company in a caring environment, and (iii) learn the skills to enhance family communication and mutual relationships. Based on the traditional Chinese values of cherishing family relationships, we introduced the general concepts of two positive psychology themes, ‘Optimism’ [25] and ‘Praise and Gratitude’ [26, 27], and their communicating values within the context of the community activities. These two themes were applied in the community activities. Table 3 shows the examples of positive family communication behaviors, which the trainees were taught to incorporate into the community activities. We also introduced the ‘5W2H’ planning skills, a mnemonic to describe the base to design programmes or activities by asking seven questions. The 5’W’s refer to ‘What to do’, ‘Why is needed’, ‘Where is the place to start the task’, ‘Who undertakes it ‘, ‘When to do it’. The 2 ‘H’s refer to ‘How to do it’ and ‘How much does it cost’. These questions intended to serve as a guide to formulate the objectives and logistics of the activities.

**Table 2:** Operational definitions of the key concepts utilized in the Learning Family Project.

Key Component	The General Concept
**Family well-being [2]**	
	It refers three domains, including family health, happiness and harmony.

	- ***Family health*** includes physical and mental health. There is a strong linkage between psychological capital, family unity and family health. - ***Family happiness*** is engendered from family activities. Spending time with family members and building connection with friends and relatives are pathways to positive family relationships and individual happiness.
	- ***Family harmony*** defined as absence of conflicts and effective communication with family members. Forbearance and spending time with family are important in forming a harmonious family.
**Learning family [1]**	
	It refers to encourage the participants joining the learning with fun activities individually and/or with their family members.
	- The learning outcomes are intended for contributing to a culture of learning in the family and community, as well as learning some knowledge, skills, and behaviour to enhance family well-being (FAMILY health, happiness and harmony).
	- The goal of the activities is to promote family communication and mutual understanding among family members.
**Neigbourhood cohesion [24]**	
	It refers the trusting network of relationships, shared values and norms of residents in a neighbourhood.
**Optimism [25]**	
	It refers to a subjective sense of positive emotion and psychological state, as well as the sense that life is worthwhile.
**Praise and Gratitude [26, 27]**	
	It refers to the expression of thankfulness and an emotional sense of appreciation.

**Table 3:** Examples of behaviours to promote in order to enhance family well-being.

**Family Health**	
	- Enjoy fresh fruits and vegetables with family members - Enjoy food with low fat, low salt, low sugar and high fibre with family members - Enjoy meals with family members and eat slowly - Spend time to walk or exercise with family members
**Family Happiness**	
	- Praise family members - Encourage family members to be optimistic when facing unhappy incidents - Share happy experiences with family members
**Family Harmony**	
	- Chat with family members - Reduce criticism towards family members - Say thank you to family members - Help to cook/prepare/clear/ wash dishes

Based on adult learning theory, we emphasized practical knowledge rather than theories. We adopted an experiential learning approach using diverse methodologies, including lectures, videos, brainstorming sessions, and small-group discussion, role play and games. We demonstrated to the trainees how to introduce the core messages (family well-being, learning with family and neighbourhood cohesion) of the project to the residents. We anticipated challenging scenarios that they might encounter (for example, a resident refusing the invitation to join the activities), and invited and offered solutions to these challenges. Training kits, teaching notes, an activities implementation guideline, and checklists were provided. The training sessions were video recorded for process evaluation.

#### Post-training phase

In the post-training phase, we continued to provide technical assistance and support for activities development through discussion, phone calls and site visits. We helped the trainees prepare tools for activity implementation, including posters and promotion pamphlets, workbooks, guideline for program implementation and souvenirs to be distributed to the community participants. We also provided incentives of HK$1000 (about US$125) per program to the Mutual Aid Committees of the housing block for the trainees together with the committees organized community activities based on the guidance received in the workshop, and delivered them in their own housing blocks, with the post-training support from the research team and social workers.

### Outcome measures

#### Reactions to training content

We asked the trainees to grade the utility of the training content in program design and implementation (one item). Responses were made on a five-point Likert scale, ranging from ‘1 = very impractical’ to ‘5 = very practical’ in the level of utility.

#### Changes in learning

We asked the trainees to indicate the extent to which they understood (perceived knowledge) the general concepts: ‘Family well-being’ (three items), ‘Learning family’ (two items), leadership skills (three items) and ‘5W2H’ planning skills (one item). Responses were made on a five-point Likert scale, ranging from ‘1 = no idea at all’ to ‘5 = know it well’ (S1 Appendix, Part A). We also asked them to assess their self-efficacy (“I am confident I have the skills”) in the application of the general concepts of ‘Learning family’ in activities design (three items) and in the use of leadership skills (four items). Responses were made on a five-point Likert scale, ranging from ‘1 = extremely incapable’ to ‘5 = highly capable’ (S1 Appendix, Part B). Finally, we evaluated their attitude towards the application of the general concept of ‘Learning family’ for enhancing family well-being (three items), and the planning skills for developing activities (one item) by asking the extent of agreement on a five-point Likert scale, ranging from ‘1 = strong disagree’ to ‘5 = strongly agree’ (S1 Appendix, Part C). A high score indicated a positive response in the above three domains of perceived knowledge, self-efficacy, and attitude. The Cronbach’s α of the above scales ranged from 0.78–0.97, indicating good to excellent internal consistency.

#### Changes in behaviors

We assessed the application of the learning by asking the trainees to indicate (i) how often they used the planning skills to develop community activities for the residents (one item); and (ii) how often they reminded the residents to learn with family members to enhance family well-being (three items). Responses were made on a five-point Likert scale, ranging from ‘1 = never’ to ‘5 = always’. The internal consistency (Cronbach’s α) was 0.89 (S1 Appendix, Part D).

#### Results of the family community activities by trainees

We additionally tested whether the community activities were effectively implemented or assisted to conduct by the trainees. We surveyed the community program participants at baseline (after the TTT but before the community programs) and one year after the start of a series of community-based family well-being activities in the housing estate. We evaluated the two levels of influence on the residents, which included their perceived personal health, family communication behaviors, family well-being, and neighborhood cohesion. Outcome–based questions were asked to assess the residents’ perception of (i) their general physical and mental health status (two items), (ii) their practice on positive family communication behaviors (eleven items), (iii) family well-being (three items), and (iv) neighborhood cohesion (five items). Responses for the general health status were made on a five-point Likert scale, ranging from ‘1 = excellent’ to ‘5 = bad’; for each item of positive family communication behaviors, were ‘Yes’ and ‘No’. A family well-being measure was created for FAMILY project, which asked residents to rate their family harmony, happiness and health. The domains of social cohesion and trust in neighborhood cohesion scale [28] were adopted to assess the residents’ perception on their neighborhood cohesion.

### Statistical analysis

Analyses were conducted using SPSS version 20.0. All significance tests were 2-sided with a 5% level of significance. Missing data of trainees who were lost to follow up, or declined to complete the questionnaire were replaced by baseline values in intention-to-treat analysis. Repeated measures analysis of variance and Friedman test were employed to compare parametric and nonparametric data at three time points, respectively; whereas paired t-test and Wilcoxon test were employed to compare parametric and nonparametric data between two time points, respectively. Data of the subgroup of trainees who had completed all assessments was also analyzed (per protocol analysis) to supplement the more conservative intention-to-treat analysis. The effect size (Cohen’s d) of the change in the outcomes was computed. This statistic reflects the magnitude of the difference and unlike significance levels is not dependent on sample size. A positive effect size indicates an increase in the standardized mean score of the outcome, while a negative effect size indicates a decrease. Effect sizes of 0.2 to < 0.5 have been described as small, 0.5 to < 0.8 as medium, and 0.8 or above as large [29]. Chi-square test was used to compare baseline characteristics between subjects who completed and those who did not complete the one-year assessment, and between subjects who joined and those who did not join the focus group interviews. All qualitative interviews were audio-taped and transcribed verbatim in Cantonese to capture every nuance of expression unique to the dialect. At least 10% of the transcripts were double-checked with the recordings. Coding was processed by two project team members, one of whom attended the interviews. Transcripts were analysed by thematic content analysis, following the guidelines recommended by Morse and Field [30]. Each transcript was analysed sentence by sentence and coded for the respondents’ meaning. The transcripts were reviewed again by another member of the project team to validate the thematic analysis and to ensure that all meaningful interview data had been analysed. Mixed Method Triangulation design was used to interrelate and interpret the qualitative and quantitative data to validate the results [31].

## Results

**Fig 2:**
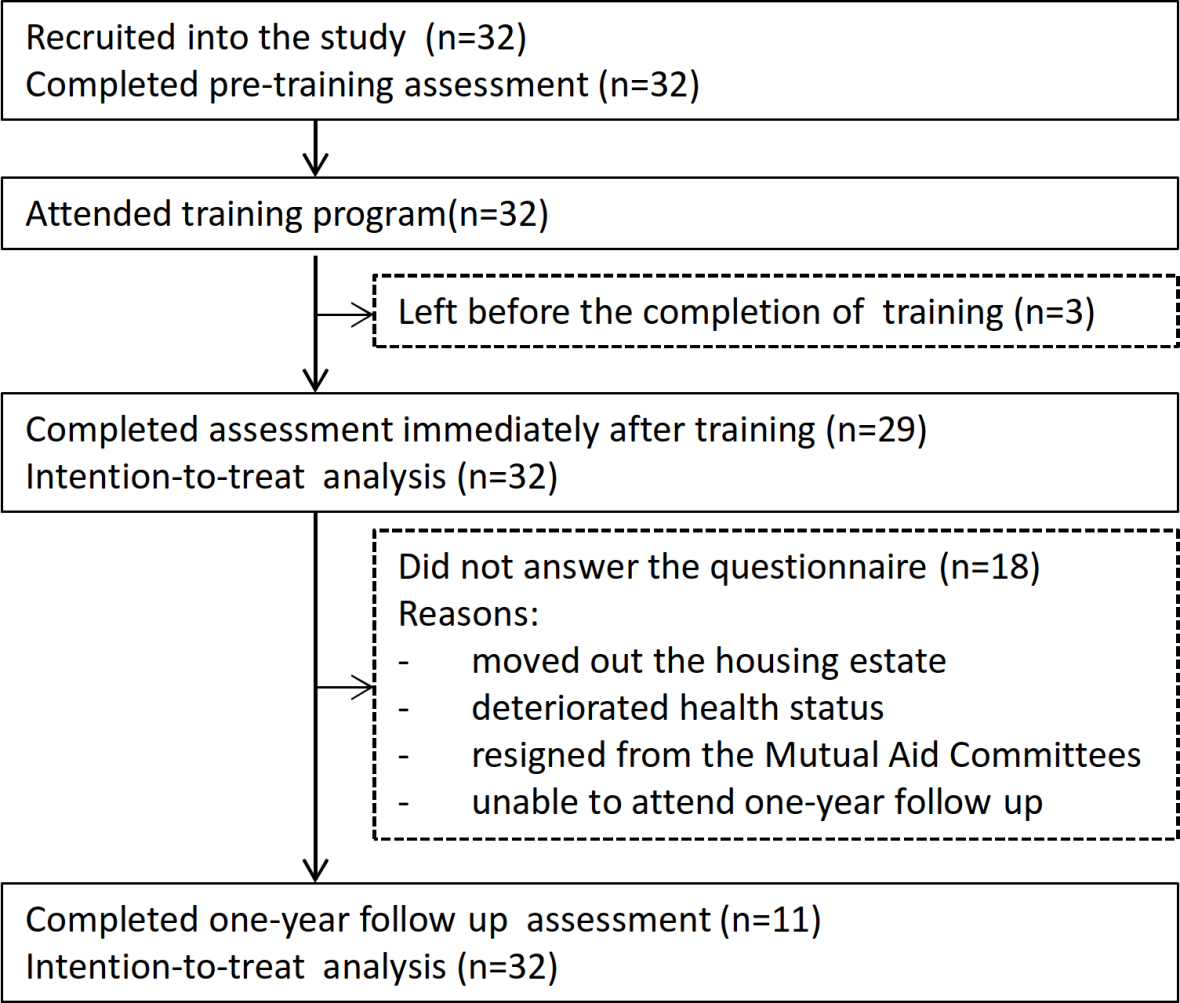
The lay health promoters’ flow diagram.

Thirty-two trainees attended the training workshop (Table 4). Three left after the training but before the evaluation, and eighteen did not answer the questionnaire at one-year assessment. Reasons for nonparticipation at follow-up included the individuals: had moved out of the housing estate, had deteriorated health, resigned from the MAC, and was unable to attend the one-year follow-up. Thirty-two questionnaires at pre-training, 29 immediately after training, and 11 at one-year follow-up were thus collected (Fig 2). There was no significant difference in the characteristics of trainees who participated in the one-year assessment and those who did not, except a significant difference was noted for the age group (< 60 and > = 60 years old) (p = 0.017) (S2 Appendix).

**Table 4:** Demographic characteristics of all trainees, those who completed one-year assessment, and those who joined the focus group interviews.

	All (n = 32)		One-year assessment (n = 11)	Focus group interviews (n = 19)
	Number (%)		Number (%)	Number (%)
**Female**	23 (72)		8 (73)	8 (42)
**Age group, years**				
18–44	5 (16)		2 (18)	2 (11)
45–59	13 (41)		1 (9)	7(36)
> = 60	14 (43)		8 (73)	10 (53)
**Education level**				
Elementary school or below	13 (41)		6 (54)	10 (53)
High school or above	19 (59)		5 (45)	9 (47)
**Duration of volunteer services in Hong Kong, years**				
< 5 years	16 (50)		6 (55)	5 (26)
≥ 5 years	16 (50)		5 (45)	14 (74)

Nineteen trainees participated in the focus group interviews after implementing a series of community activities, at approximately one and a half years after training (Table 4). There was no significant difference in the characteristics of trainees who participated in the focus group interviews and those who did not, except a significant difference was noted for the duration of volunteer services in Hong Kong (<5 years and > = 5 years) (p = 0.012) (S2 Appendix).

### Reactions to training content

Almost 70% of the trainees rated the utility of the training content as ‘practical’ or ‘very practical’ both immediately after training and at one-year assessment. The qualitative results corroborated the quantitative finding regarding satisfaction with the training. The trainees joined the focus group interviews gave positive responses to the training content.

*“The concept of 3Hs (family health*, *family happiness and family harmony) is meaningful*. *It is easy to understand*.*”* (woman, ages 55–64 years)

*“I liked the concept of the 3Hs (family health*, *family happiness and family harmony)*.*”* (woman, aged 25–34 years)

### Changes in learning

Table 5 shows significant increases in perceived knowledge, self-efficacy, and attitude in relation to the general concepts of ‘Family well-being’, ‘Learning family’, leadership and planning skills with small to large effect sizes (Cohen’s d: 0.4–1.2), immediately after training. The effects on the perceived knowledge of leadership and planning skills, as well as on attitude towards the application of the general concept of ‘Learning family’ to enhance family well-being were sustained to one year after training with small to medium effect sizes (Cohen’s d: 0.3–0.6). Per-protocol analysis showed similar findings but with greater effect sizes at one year (S3 Appendix).

**Table 5:** Trainees’ perceived knowledge, self-efficacy, attitude and practice on each component of Learning Families Project: Intention-to-treat analysis.

		Pre-training	Immediately after training	1- year	Difference between	
	n = 32				Pre-training and Immediate after training	Pre-training and 1 year
		Mean score ± SD			Cohen’s d^e^/ p-value	
**Perceived knowledge of the general concepts of** ^a^						
-	Family well-being ^#^	4.1 ± 0.6	4.3 ± 0.5	4.2 ± 0.6	0.52 / < 0.01**	0.28/ 0.125
-	‘Learning family’ to enhance family well-being ^#^	3.6 ± 0.8	4.0 ± 0.6	3.7 ± 0.8	0.48 / < 0.05*	0.19 / 0.301
-	Leadership skills ^# # #^	3.1 ± 0.7	3.7 ± 0.7	3.4 ± 0.7	0.88 / < 0.001***	0.38 / < 0.05*
-	Planning skills to develop activities ^# # #^	2.0 ± 0.9	3.6 ± 1.0	2.5 ± 1.0	1.24 / < 0.001***	0.60 / < 0.01**
**Self–efficacy in relation to**^b^						
-	Engaging residents in activities with their family members	3.1 ± 0.6	3.4 ± 0.6	3.3 ± 0.7	0.39 / < 0.05*	0.35 / 0.061
-	Applying leadership skills in practice	3.0 ± 0.8	3.4 ± 0.8	3.2 ± 0.7	0.40 / < 0.05*	0.29 / 0.106
**Attitude towards the program**^c^						
-	The application of ‘Learning family’ concept can enhance residents’ family well-being ^#^	3.8 ± 0.7	4.1 ± 0.6	4.0 ± 0.8	0.55/ < 0.01***	0.41 / < 0.05*
-	The application of planning skills can help the development of activities for the residents ^# # #^	2.8 ± 1.1	4.1 ± 0.8	3.1 ± 1.1	1.03 / < 0.001***	0.30 / 0.095
**Practice**^d^						
-	Applying the general concept of ‘Learning family’ to enhance residents’ family well-being	3.1 ± 1.2	—-	3.4 ± 1.3	—-	0.24 / 0.118
-	Using planning skills to develop activities	1.8 ± 1.1	—-	2.3 ± 1.3	—-	0.40 / < 0.05*

Number of questions: perceived knowledge (10 items), attitude towards the practice (4 items), and self-efficacy (7 items)

^a^5-point Likert scale: 1 = no idea at all; 2 = no idea; 3 = neutral; 4 = understand; 5 = know it well

^b^ 5-point Likert scale: 1 = incapable at all; 2 = incapable; 3 = neutral; 4 = capable; 5 = highly capable

^c^ 5-point Likert scale: 1 = strongly disagree; 2 = disagree; 3 = neutral; 4 = agree; 5 = strongly agree

^d^ 5-point Likert scale: 1 = never; 2 = rare; 3 = sometimes; 4 = occasionally; 5 = always

Repeated measures analysis of variance was used to compare the scores at three time points

^#^ p<0.05

^# #^p<0.01

^# # #^p<0.01

Paired t-test was used to compare the mean at two time points

* p value <0.05

** p value <0.01

*** p value <0.01

^e^ Effect size (Cohen’s d): small = 0.20, medium = 0.50 and large = 0.80

### Changes in behaviors

At one-year assessment, there was a significant increase in the application of the planning skills in developing activities with small effect size (Cohen’s d: 0.4), but not in the application of ‘Learning family’ concept to enhance family well-being (Table 5).

At the focus group interview, the trainees reported that they had actively participated in the community programme and had tried to recruit the “hard to reach” residents. Trainees also indicated enhanced relationships with their neighbours.

*“We enjoyed the door-to-door visits*, *which were intended to promote the activities (to other households) on each floor*. *Because of these efforts we have become well acquainted with residents (of the estate)*.*”* (man, aged 55–64 years)

*“I enjoy that I can now chat with the residents and help them when they are in need*.*”* (man, aged 55–64 years)

*“The change is great*. *There are several elderly residents*, *who sit in the public areas during the day for leisure*, *and now they smile and greet me when we meet*.*”* (woman, aged 65+ years)

The working relationships among different parties were good, but there was a room to improve the communication among the different working parties.

*“They (the staff of CFSC) are very good*. *If not*, *we (members of MAC) will not help implement the program*.*”* (woman, 65+ years)

*“We (MACs) were asked to gather for the kick-off ceremony*, *but no specific instruction was given about what we should expect*. *I think it would have been helpful to have more details about the programme earlier*.*”* (woman, 65+ years)

Trainees provided information about the difficulties they encountered during the program: the questionnaires were lengthy and repetitive; and the recruitment of male community activity participants was difficult. Integrating the quantitative and qualitative findings, the trainees’ enhanced competence helped the implementation of community programs and the promotion of and the neighborhood cohesion in their community.

*“The questionnaire has many pages*. *Similar questions were asked again and again*. *It is repetitive*.*”* (woman, 65+ years)

*“It was really difficult to motivate the men (to join the activities)*. *The men in this estate… may be it is a cultural issue……*. *Even those who are my neighbours living in the same block*, *I could not motivate them*.*”* (man, 55–64 years)

### Results of the family community activities by trainees

After training, the trainees were guided by the research team of HKU-SPH and social workers of CFSC, implemented 14 ‘Learning programs’ for 208 participants in their own housing blocks. They also assisted the social workers to conduct a series of fieldwork recruitment of residents and community activities. The communities activities included ten promotion programmes for 670 participants, 24 ‘Resident training programmes’ for 980 participants, and six “Love your family eco-farm” visits for 365 participants, kick–off and recognition ceremonies for nearly 1000 participants, and two household surveys (more than 1000 participants in each survey).

A total of 1167 and 1323 questionnaires were collected from the residents in Tsui Ping (South) Estate in March 2011 and March 2012, respectively. Two-thirds of respondents were women. Over 90% of them had been living in Hong Kong for over seven years; 30% of them were aged 65 years or older, and nearly 40% of them had received only a primary school education or less. Physical and mental health, family communication behaviours and neighbour cohesion were improved significantly at one-year follow-up survey, compared to the baseline. However, only a small insignificant increase in the family well-being score was found. The details were shown in the report of the Learning Families Project [1].

We also compared the findings from Shun Tin Estate, which acted as a control estate. Shun Tin Estate is a government low rent housing estates in Kwun Tong, which located about 2.6 km apart from (Tsui Ping (South) Estate), and are well separated by busy main roads. The residents of both estates were with similar socio-economic background. Both 1108 residents participated in the baseline and follow-up survey. The intervention estate (Tsui Ping (South) Estate) showed more increase in neighbourhood cohesion, compared to control estate (Shun Tin Estate). The details have been presented in a report to the funding organization and was reported in our sister paper and the report of the Learning Families Project [1].

## Discussion

To our knowledge, this is the first TTT workshop to teach lay volunteers (trainees) to be lay health promoters, who subsequently implemented and assisted the delivery of family well-being activities within their community. The two two-hour TTT workshop enhanced trainees’ competence on incorporating the general concepts of specific positive psychology themes (Optimism’ [25] and ‘Praise and Gratitude’ [26, 27] and learning with family into community-based family well-being activities, immediately after training. The effects on knowledge and attitude towards practice were sustained to one year.

Our TTT workshop had different characteristics from others designed to train lay health promoters reported in the literature. From our knowledge, most TTT workshops for lay health promoters were conducted over a longer period of time: eight 2-hour sessions to promote physical activity [9, 10], 3 to 4 days for HIV peer educators [17], and nine 3-hour sessions with two full-day sessions on research knowledge for community leaders [32]. The TTT workshops were also used to enhance family well-being in the community, which have been conducted for health professionals. For examples: a 5-day national training program was conducted for 120 rural nurses in Australia [33], and a 5-module training program (2 to 3 days each module) was implemented for primary health care workers in Canada [34].

Our TTT workshop was designed as a brief two two-hour session workshop, which minimised the burden of the social and health resources, enhanced the recruitment of trainees, and ensured the completion of training. Many of our trainees had low education level (40% received primary education or below), and were older (31% aged over 65 years). After considering the needs of the community and trainees’ literacy, the training curriculum was developed with three aspects: (i) the scope and depth of the content, (ii) the structure of activities, and (iii) the training methods. The content was specific to enhance family well-being, easy to understand, and applicable to direct practice. We ensured the construction of the workshop to have adequate time to cover the content to the desired level of depth and to link with the activities to be delivered. We taught the topics from fundamental (the general concepts of the key components used in the project, for example, positive psychology themes) to more refined applications (the application of suggested positive family communication to enhance family well-being into the community activities) (Tables 2 and 3). Various training methods were used to promote learning and retention, such as discussion for better understanding and experiential exercises for developing self-awareness. We also provided the post-training technical assistance and support, which aimed to empower the trainees’ competence and performance on program implementation. The post-training assistance and support have been considered as major factors for the successful implementation of community activities [17, 35]. In the meantime, the trainees gained confidence about using their existing social networks and engaging the residents to participate in the community activities. The empowerment of community residents created an environment for change in the residents’ attitudes and behavior [36], and enhanced family well-being and neighborhood cohesion.

The training workshop was assessed systematically by a four-level model-based evaluation [20]. The findings showed that the perceived knowledge, self-efficacy and attitude towards the topics covered significantly improved after training, which were similar to the findings of our previous TTT workshops for social workers to implement family well-being programs [37, 38]. The effects on knowledge and attitude towards practice were sustained to one year, but not the self-efficacy. Knowledge is a prerequisite for changing behavior [39, 40]. The intervention effects diminished at one year, which might be because the stimulus for change is gone and the high attrition rate (more than half of the trainees did not complete one-year assessment) [41]. We assumed no improvement in learning on those trainees who did not complete the questionnaire in the intention analysis, which was the more conservative approach. We also analysed the data with the per-protocol analysis (only included the data of those trainees completed one year assessment) with greater effect size. Trainees with older age and longer experience in volunteer community service were noted to be more willing on completing one year assessment and joining the focus group interview, respectively. It may be explained by that older trainees (for example: retirees), who may be more time, and could be more committed to the project and be more readily motived than younger volunteers [42].

Our project maximized existing community resources. We recruited resident leaders within the community to be lay health promoters. These individuals shared language, socio-economic status, and life experience with those they would serve, and had well-established ties within the community [43]. We engaged resident leaders in the needs assessment of the earliest phase of the project, which laid the groundwork for community participation and set the stage for collaboration between the research team and resident leaders. By providing the resident leaders an opportunity to voice their opinions, we were able to foster their sense of ownership of the project. Furthermore, they were experts with regards to the needs and interests of their own community. By incorporating their suggestions into the programs, we increased the likelihood that the programs we developed were meaningful and relevant to the targeted population. We successfully used the train-the–trainer approach to build the community workforce to deliver services and messages to the public in a more efficient, effective, and cost-effective way.

There were several limitations to our study. First, validated questionnaires were not available; we used outcome-based questions to evaluate the effectiveness of the workshop. We measured the perceptions such as perceived knowledge, not actual information or skills acquired. Perception can be influenced by the individual’s personality and self-perception [44]. Second, the sample size was small, the resident leaders we included might not be representative of other leaders and volunteers. Third, our study had no control group; we might have under- or over-estimated the effect of the training workshop by the regression to mean and social desirability bias. Objective measures or examinations of specific knowledge and skills, and a control group of trainees who do not receive the intervention program, and a large sample size would provide stronger evidence in future studies.

## Conclusions

Our study demonstrated a successful community-academic research partnership. It stands as a testimony to lay individuals, including those from a culture, age, and education where psychological concepts are not part of everyday discourse can be trained briefly and mobilized to promote family well-being efficiently and effectively. Training for lay health promoters in public health may offer a model for cost-effective training and interventions to benefit and large numbers of service targets. Our TTT could serve as a practical example of development and evaluation of training programs for lay personnel to be lay health promoters.

## Supporting information

S1 Appendix Questionnaire for the training workshop of Learning Families Project. (DOC)

S2 Appendix Demographic characteristic of the trainees completed the one-year assessment and those who did not; and trainees participated in the focus group interviews and those who did not. (DOC)

S3 Appendix Trainees’ perceived knowledge, self-efficacy, attitude and practice on each component of Learning Families Project: Per-protocol analysis. (DOC)
