# Supplementary material for: Development and evaluation of a training workshop for lay health promoters to implement a community-based intervention program in a public low rent housing estate: The Learning Families Project in Hong Kong
Source: PLoS One. 2017 Aug 25;12(8):e0183636. doi: 10.1371/journal.pone.0183636 (PMC5571957; doi:10.1371/journal.pone.0183636)
Supplement: S1 Appendix — (DOC) [file pone.0183636.s001.doc]

**S1 Appendix Questionnaire for training workshop of Learning Families Project**

| **Part A: Please rate your extent of understandability on the following components** | | | | | | | | | | | | | | | | | | | |
| --- | --- | --- | --- | --- | --- | --- | --- | --- | --- | --- | --- | --- | --- | --- | --- | --- | --- | --- | --- |
| ***Family well-being*** | | | | **No idea at all** | | | | **No idea** | | | **Neutral** | | | | **Know it** | | | **Know it well** | |
| 1.1 Family health includes physically and mentally healthy. There is a strong linkage between psychological capital, family unity and family health. | | | | ➀ | | | | ➁ | | | ➂ | | | | ➃ | | | ➄ | |
| 1.2 Family happiness is engendered from family activities. Spending time with family members and building connection with friends and relatives will lead to happiness. | | | | ➀ | | | | ➁ | | | ➂ | | | | ➃ | | | ➄ | |
| 1.3 Family harmony means absence of conflicts and effective communication with family members. Forbearance and spending time with family are important in forming a harmonious family. | | | | ➀ | | | | ➁ | | | ➂ | | | | ➃ | | | ➄ | |
| ***Learning Family*** | | | |  | | | |  | | |  | | | |  | | |  | |
| 1.4 The general concept of “Learning family is to join the fun activities with their family members and learning some behavior to enhance family well-being. | | | | ➀ | | | | ➁ | | | ➂ | | | | ➃ | | | ➄ | |
| 1.5 The advantage of being a learning family is to enhance the communication and relationship between family members | | | | ➀ | | | | ➁ | | | ➂ | | | | ➃ | | | ➄ | |
| ***Leadership and ‘5W2H’ planning skills*** | | | |  | | | |  | | |  | | | |  | | |  | |
| 1.6 Decision making methods | | | | ➀ | | | | ➁ | | | ➂ | | | | ➃ | | | ➄ | |
| 1.7 The characteristics of a leader | | | | ➀ | | | | ➁ | | | ➂ | | | | ➃ | | | ➄ | |
| 1.8 The functions of a leader | | | | ➀ | | | | ➁ | | | ➂ | | | | ➃ | | | ➄ | |
| 1.9 ‘5W2H’ planning skills | | | | ➀ | | | | ➁ | | | ➂ | | | | ➃ | | | ➄ | |
| **Part B: Please rate your comment on the capability on the following components** | | | | | | | | | | | | | | | | | | | |
| ***Family well-being*** | **Incapable at all** | | | | **Incapable** | | | | | **Neutral** | | | | **Capable** | | | **Highly capable** | | |
| 2.1 To engage residents in activities with their family members to enhance family health | ➀ | | | | ➁ | | | | | ➂ | | | | ➃ | | | ➄ | | |
| 2.2 To engage residents in activities with their family members to enhance family happiness | ➀ | | | | ➁ | | | | | ➂ | | | | ➃ | | | ➄ | | |
| 2.3 To engage residents in activities with their family members to enhance family harmony | ➀ | | | | ➁ | | | | | ➂ | | | | ➃ | | | ➄ | | |
| ***Leadership skills*** |  | | | |  | | | | |  | | | |  | | |  | | |
| 2.4 To apply different group decision method | ➀ | | | | ➁ | | | | | ➂ | | | | ➃ | | | ➄ | | |
| 2.5 To design activities for residents | ➀ | | | | ➁ | | | | | ➂ | | | | ➃ | | | ➄ | | |
| 2.6 To implement activities for residents | ➀ | | | | ➁ | | | | | ➂ | | | | ➃ | | | ➄ | | |
| 2.7 To allocate job responsibilities to team members according to their strengths | ➀ | | | | ➁ | | | | | ➂ | | | | ➃ | | | ➄ | | |
| **Part C: Please select to what extent you agree with the following sentences in relation to** | | | | | | | | | | | | | | | | | | | |
| ***Family well-being*** | | **Strongly disagree** | | | | **Disagree** | | | **Neutral** | | | | **Agree** | | | **Strongly Agree** | | | |
| 3.1 Learning with family is a good method to enhance family health | | ➀ | | | | ➁ | | | ➂ | | | | ➃ | | | ➄ | | | |
| 3.2 Learning with family is a good method to enhance family happiness | | ➀ | | | | ➁ | | | ➂ | | | | ➃ | | | ➄ | | | |
| 3.3 Learning with family is a good method to enhance family harmony | | ➀ | | | | ➁ | | | ➂ | | | | ➃ | | | ➄ | | | |
| ***‘5W2H’ planning skills*** | |  | | | |  | | |  | | | |  | | |  | | | |
| 3.4 ‘5W2H’ planning skills’ is a worthwhile practice | | ➀ | | | | ➁ | | | ➂ | | | | ➃ | | | ➄ | | | |
| **Part D: Please rate your extent of practice with the following applications** | | | | | | | | | | | | | | | | | | | |
| ***Family well-being*** | **Never** | | **Seldom** | | | | **Occasional** | | | | | **Sometimes** | | | | | | | **Always** |
| 3.1 In the past 4 weeks , how often you apply the general concept of ‘Learning family’ to enhance residents’ family health | ➀ | | ➁ | | | | ➂ | | | | | ➃ | | | | | | | ➄ |
| 3.2 In the past 4 weeks , how often you apply the general concept of ‘Learning family’ to enhance residents’ family happiness | ➀ | | ➁ | | | | ➂ | | | | | ➃ | | | | | | | ➄ |
| 3.3 In the past 4 weeks , how often you apply the general concept of ‘Learning family’ to enhance residents’ family harmony | ➀ | | ➁ | | | | ➂ | | | | | ➃ | | | | | | | ➄ |
| ***‘5W2H’ planning skills*** |  | |  | | | |  | | | | |  | | | | | | |  |
| 3.4 ‘ In the past 4 weeks , how often you use ‘5W2H’ planning skills to develop activities | ➀ | | ➁ | | | | ➂ | | | | | ➃ | | | | | | | ➄ |
