# Supplementary material for: Development and evaluation of a training workshop for lay health promoters to implement a community-based intervention program in a public low rent housing estate: The Learning Families Project in Hong Kong
Source: PLoS One. 2017 Aug 25;12(8):e0183636. doi: 10.1371/journal.pone.0183636 (PMC5571957; doi:10.1371/journal.pone.0183636)
Supplement: S2 Appendix — (DOC) [file pone.0183636.s002.doc]

**S2 Appendix Demographic characteristic of the trainees completed the one-year assessment and those who did not; and trainees participated in the focus group interviews and those who did not**

|  | **One-year assessment** | | | | | **Focus group interviews** | | |
| --- | --- | --- | --- | --- | --- | --- | --- | --- |
|  | **Completed**  **(n=11)**  **Number (%)** | **Did not complete**  **(n=21)**  **Number (%)** | | | **p value** | **Participated**  **(n=19)**  **Number (%)** | **Did not participate**  **(n=13)**  **Number (%)** | **p value** |
| **Gender** |  |  | | |  |  |  |  |
| Female | 8 (73) | 15 (71) | | | 0.958 | 11 (58) | 9 (69) | .515 |
| Male | 3 (27) | 6 (29) | | |  | 8 (42) | 4 (31) |  |
|  |  |  | | |  |  |  |  |
| **Age group, years** |  |  | | |  |  |  |  |
| <60 | 3 (27) | 15 (71) | | | 0.017* | 9 (47) | 9 (69) | 0.221 |
| >=60 | 8 (73) | 6 (29) | | |  | 10 (53) | 4 (31) |  |
|  |  |  | | |  |  |  |  |
| **Education level** |  |  | | |  |  |  |  |
| Elementary school or below | 6 (55) | 7 (33) | | | 0.246 | 10 (53) | 3 (23) | 0.09 |
| High school or above | 5 (45) | 14 (67) | | |  | 9 (47) | 10 (77) |  |
|  |  |  | | |  |  |  |  |
| **Duration of volunteer services,**  **years** |  | | | | |  |  |  |
| < 5 years | 6 (55) | 10 (48) | | 0.710 | | 5 (26) | 11 (85) | 0.012* |
| ≥ 5 years | 5 (45) | 11 (52) |  | | | 14 (74) | 2 (15) |  |

Chi-square test was used to compare the difference between two groups, * p value <0.05
