# Supplementary material for: Development and evaluation of a training workshop for lay health promoters to implement a community-based intervention program in a public low rent housing estate: The Learning Families Project in Hong Kong
Source: PLoS One. 2017 Aug 25;12(8):e0183636. doi: 10.1371/journal.pone.0183636 (PMC5571957; doi:10.1371/journal.pone.0183636)
Supplement: S3 Appendix — (DOC) [file pone.0183636.s003.doc]

**S3 Appendix Trainees’ perceived knowledge, self-efficacy, attitude and practice on each component of Learning Families Project: Per-protocol analysis**

|  |  |  |  |  | **Difference between** | |
| --- | --- | --- | --- | --- | --- | --- |
|  | **n = 11** | **Pre-training** | **Immediately after training** | **1- year** | **Pre-training and**  **Immediate after training** | **Pre-training and**  **1 year** |
|  | | **Mean score ± SD** | | | **Cohen’s d^e^/ p-value** | |
| **Perceived knowledge of** **the general concepts of** ^a^ | |  | | |  | |
| - | Family well-being | 4.1 ± 0.5 | 4.4 ± 0.6 | 4.4 ± 0.4 | 0.42 / < 0.05* | 0.33 / 0.119 |
| - | ‘Learning family’ to enhance family well-being | 3.8 ± 0.5 | 4.2 ± 0.6 | 4.0 ± 0.5 | 0.48 / < 0.05* | 0.19 / 0.370 |
| - | Leadership skills ^#^ | 3.2 ± 0.8 | 3.8 ± 0.7 | 3.8± 0.6 | 0.52 / < 0.05* | 0.39 / 0.064 |
| - | Planning skills to develop activities ^# #^ | 1.7 ± 0.8 | 3.8 ± 1.1 | 3.4 ± 0.7 | 0.60 / < 0.01** | 0.61 / < 0.01* |
| **Self–efficacy in relation to**^b^ | |  |  |  |  |  |
| - | Engaging residents in activities with their family members | 3.3 ± 0.5 | 3.5 ± 0.5 | 3.8 ± 0.7 | 0.25 / 0.234 | 0.38 / 0.078 |
| - | Applying leadership skills in practice | 3.0 ± 1.0 | 3.6 ± 0.8 | 3.7 ± 0.5 | 0.33 / 0.125 | 0.35 / 0.097 |
| **Attitude towards the programme**^c^ | |  |  |  |  |  |
| - | The application of ‘Learning family’ concept can enhance residents’ family well-being ^#^ | 3.8 ± 0.7 | 4.2 ± 0.6 | 4.5 ± 0.5 | 0.40 / 0.061 | 0.44 / < 0.05* |
| - | The application of planning skills can help the development of activities for the residents ^#^ | 3.1 ± 1.2 | 4.1 ± 0.7 | 3.9 ± 0.5 | 0.51 / <0.05* | 0.35 / 0.105 |
| **Practice** ^d^ | |  |  |  |  |  |
| - | Applying the general concept of ‘Learning family’ to enhance residents’ family well-being | 2.9 ± 1.0 | ---- | 3.7 ± 1.2 | ---- | 0.33/ 0.123 |
| - | Using planning skills to develop activities | 1.8 ± 1.3 | ---- | 3.2 ± 1.1 | ---- | 0.45 / < 0.05* |

Number of questions: perceived knowledge (10 items), attitude towards the practice (4 items), and self-efficacy (7 items),

^a^5-point Likert scale: 1 = no idea at all; 2 = no idea; 3 = neutral; 4 = understand; 5 = know it well

^b^ 5-point Likert scale: 1 = incapable at all; 2 = incapable; 3 = neutral; 4 = capable; 5 = highly capable

^c^ 5-point Likert scale: 1 = strongly disagree; 2 = disagree; 3 = neutral; 4 = agree; 5 = strongly agree

^d^ 5-point Likert scale: 1 = never; 2 = rare; 3 = sometimes; 4 = occasionally; 5 = always

Friedman test was used to detect the difference at three time points; ^#^ p value <0.05, ^# #^p value < 0.001,

Wilcoxon test was used to compare the mean at two time points; * p value <0.05, **p value < 0.01

^e^ Effect size (Cohen’s d): small = 0.20, medium = 0.50 and large = 0.80
